# Association Between the Hemoglobin Glycation Index and the Risk of Delirium in Stroke Patients Admitted to the Intensive Care Unit: A Retrospective Study Based on the MIMIC‐IV Database

**DOI:** 10.1002/brb3.71768

**Published:** 2026-09-23

**Authors:** Yuwen Wang, Xu Yang, Yong Yin

**Affiliations:** ^1^ Department of Rehabilitation Medicine The Affiliated Hospital of Yunnan University Kunming Yunnan China

**Keywords:** hemoglobin glycation index, ICU, MIMIC‐IV, poststroke delirium, stroke

## Abstract

**Background:**

The hemoglobin glycation index (HGI) has been introduced to reflect discrepancies between chronic baseline glucose and acute stress‐induced glucose changes. HGI has been linked to neurological recovery and poor prognosis among stroke patients. However, its role in poststroke delirium (PSD) remains to be clarified. This study aimed to evaluate the association between HGI levels and the risk of PSD during intensive care unit (ICU) stay in stroke patients.

**Methods:**

Patient data were extracted from MIMIC‐IV v3.1. HGI was derived using linear regression analysis based on the relationship between HbA1c levels and fasting plasma glucose (FPG) concentrations. Logistic regression analysis was used to assess the association between HGI and the risk of PSD during ICU stay. The restricted cubic spline (RCS) method was applied to investigate possible nonlinear relationships. Subgroup and interaction analyses were performed to evaluate the robustness of the results.

**Results:**

A total of 2247 patients were included. Of these individuals, 707 developed PSD during ICU stay. Logistic regression indicated that a low HGI value was significantly associated with an elevated risk of PSD. RCS analysis suggested a nonlinear association. The inflection point was identified at HGI = −0.11. For HGI values ≤ −0.11, a one‐unit increase in HGI corresponded to a 30.9% lower risk of PSD (OR = 0.691, 95% CI: 0.554–0.859, *p* < 0.001).

**Conclusions:**

Lower HGI levels were significantly associated with an elevated risk of PSD during ICU stay among severely ill stroke patients. HGI may be a valuable indicator for detecting patients at high risk of PSD.

## Graphical abstract

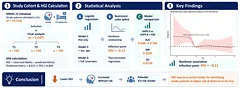

## 1 Introduction

Stroke is a major global cause of mortality and disability. It includes ischemic and hemorrhagic subtypes (Martin et al. 2024). In the last 20 years, the incidence of stroke has risen by 70%, prevalence by 85%, and mortality by 43% (GBD 2021 Stroke Risk Factor Collaborators 2024). The high incidence, disability, mortality, and recurrence of stroke lead to a significant economic burden for individuals, families, and society (Tu et al. 2023). In the management of acute stroke, early diagnosis, timely treatment, rehabilitation, and recurrence prevention are essential for saving as much brain tissue as possible and minimizing neurological damage (Tao et al. 2020). Despite aggressive care, many survivors experience neurological dysfunction. Delirium remains one of the most frequent complications. It is characterized by an abrupt disturbance in consciousness and cognitive function, and is particularly prevalent among hospitalized old patients (Zhang et al. 2024). Meta‐analyses suggest that approximately 25% of acute stroke patients develop poststroke delirium (PSD) (Shaw et al. 2019). Nonetheless, risk factors for PSD remain inconclusive.

Diabetes mellitus frequently coexists with stroke. Patients with diabetes mellitus have a significantly elevated risk of stroke compared to those without diabetes mellitus (Lau et al. 2019; Zou et al. 2023; Jerkins and Bell 2025). Single admission glucose measurements are insufficient to reflect long‐term glycemic status. HbA1c reflects mean blood glucose concentration over the past 3 months and thus can systematically assess the efficacy of blood glucose control. However, HbA1c is different from other glycemic indicators. Its measurement reliability can be influenced by red blood cell factors, enzymatic abnormalities, and hemoglobin (Hb) gene variants. These influences are more pronounced in individuals with normal glucose levels (Brown et al. 2005; Khera et al. 2008; Malka et al. 2016). Therefore, the hemoglobin glycation index (HGI) is developed to quantify the difference in the link between HbA1c and plasma glucose concentrations (Hempe et al. 2015). HGI offers unique value in evaluating glycemic status, providing a precise measure for research and clinical practice (Hempe and Hsia 2022). Previous studies have identified HGI as an independent marker of adverse cardiovascular events, including heart failure and coronary artery disease (Liu et al. 2025; Wei et al. 2024; W. Cheng et al. 2024). Evidence on the influence of HGI on stroke outcomes remains limited.

This study aimed to investigate the association between HGI and PSD in stroke patients during intensive care unit (ICU) stay. The findings may provide insights into the early identification of high‐risk patients and improvement of clinical outcomes.

## 2 Methods

### 2.1 Data Source

Retrospective data from the MIMIC‐IV v3.1 (released in October 2024) were used in this study. It is a large, open‐access critical care dataset constructed based on data at Beth Israel Deaconess Medical Center (Boston, USA). It includes data from over 65,000 individuals hospitalized in the ICU and over 200,000 individuals in the emergency department (ED). To access the data, the author Yuwen Wang completed the National Institutes of Health (NIH) training course on protecting human research participants and passed the Collaborative Institutional Training Initiative (CITI) exam (Record ID 68496220). Informed consent was waived owing to de‐identified health information.

### 2.2 Study Population

**FIGURE 1:**
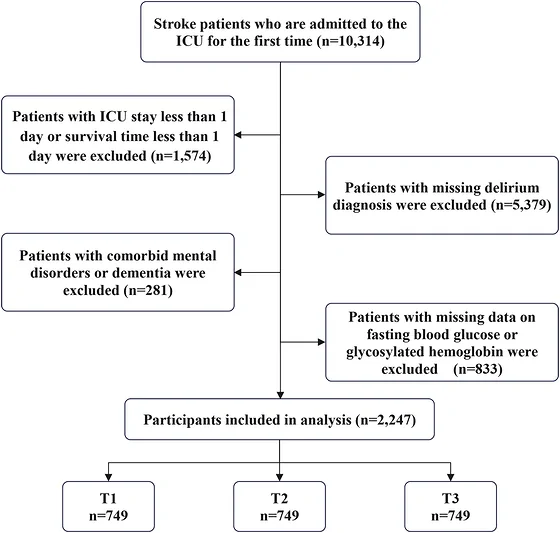
Flow of included patients through the trial.

We initially identified 10,314 ICU patients with stroke in the database. The exclusion criteria were as follows: (Martin et al. 2024) ICU stay under 24 h; (GBD 2021 Stroke Risk Factor Collaborators 2024) missing or incomplete delirium data; (Tu et al. 2023) no HbA1c or fasting plasma glucose (FPG) measurement within 24 h of admission; (Tao et al. 2020) prior psychiatric disorders or dementia. Eventually, 2247 patients were included in this study. Individuals were divided into three groups based on tertiles of HGI (Figure 1).

### 2.3 Data Extraction

Data were extracted using PostgreSQL (version 16.8) via structured query language. The following variables were included: (Martin et al. 2024) demographics: age, sex, and ethnicity; (GBD 2021 Stroke Risk Factor Collaborators 2024) medical history: hypertension, diabetes, sepsis, schizophrenia, and dementia; (Tu et al. 2023) vital signs: heart rate, respiratory rate, and oxygen saturation; (Tao et al. 2020) laboratory parameters: FPG, Hb, HbA1c, high‐density lipoprotein, low‐density lipoprotein, triglycerides, total cholesterol, international normalized ratio (INR), platelets, white blood cells, serum albumin, red blood cells, aspartate/alanine aminotransferase, D‐dimer, serum calcium/sodium/potassium, and serum creatinine; (Zhang et al. 2024) interventions: mechanical ventilation and continuous renal replacement therapy; (Shaw et al. 2019) medications: aspirin, furosemide, insulin, statins, and fibrates; (Lau et al. 2019) hospitalization and outcomes: ICU length of stay (ICU LOS), 30‐/90‐/ 365‐day mortality; (Zou et al. 2023) severity scores: Glasgow Coma Scale (GCS) and Simplified Acute Physiology Score II (SAPSII) (Cheng et al. 2024). All values were obtained from the first measurement after ICU admission and before any therapeutic interventions. Variables with > 20% missing values were removed. For participants with ≤ 20% missing values, multiple imputation was performed using the “mice” package in R based on a random forest algorithm, with five imputations. The imputation model included all variables in the final multivariable models as well as the outcome variable and key predictors to minimize bias. Missing data were assumed to be missing at random, conditional on observed covariates. The missingness proportion for each variable is presented in Table S1.

### 2.4 Outcome Definition

The main outcome was PSD, defined as delirium occurring during ICU stay in stroke patients. Delirium was evaluated at the bedside at least twice per day via the Confusion Assessment Method for the patients in ICU (CAM‐ICU). Two trained nurses independently evaluated patients for the core CAM‐ICU characterizations: abrupt onset, impaired attention, disorganized thought, and altered level of consciousness (Miranda et al. 2023; Stollings et al. 2021). Each patient was assessed once in the morning shift (approximately 7–9 a.m.) and once in the evening shift (approximately 7–9 p.m.) daily. Level of consciousness was assessed using the Richmond Agitation‐Sedation Scale (RASS), and CAM‐ICU assessments were performed only for patients with RASS ≥ −3 (Brummel et al. 2024). Delirium was diagnosed when both nurses reached an agreement. Disagreements, if any, were resolved by a third reviewer. CAM‐ICU assessments that were missing or incomplete were not included in the analysis.

### 2.5 HGI Calculation

A linear regression model linking FPG to HbA1c was built with this study cohort. Based on the equation (HbA1c (predicted) = 0.013 × FPG + 4.804) (Table S2), HGI was defined as the difference between observed and estimated HbA1c levels (Hempe et al. 2015). Detailed regression statistics, including regression coefficients, standard errors, *t*‐values, *p*‐values, and the coefficient of determination (*R* ^2^), are presented in Table S2.

### 2.6 Statistical Analysis

Participants were divided into three groups according to the tertiles of HGI: T1 (*n* = 749, HGI ≤ −3.92), T2 (*n* = 749, −3.92 < HGI ≤ 0.14), and T3 (*n* = 749, HGI > 0.14). Categorical variables were expressed as frequencies (*n*) and percentages (%) and were compared using Pearson's chi‐square test. Continuous variables were expressed as medians with interquartile ranges (IQRs) and were compared using the Kruskal–Wallis *H* test. The multicollinearity among all candidate variables was assessed using the variance inflation factor (VIF). Variables with a VIF of five or higher, suggesting moderate or higher multicollinearity, were excluded to ensure the stability and reliability of regression coefficients. VIF values for all candidate variables are presented in Table S3. The association of HGI with the risk of PSD was evaluated using logistic regression (reference: T2). Model 1 was only adjusted for HGI index. Model 2 was additionally adjusted for sex and age. Model 3 was further adjusted for race, vital signs (oxygen saturation, respiratory rate, and heart rate), comorbidities (hypertension, sepsis, diabetes), medication use (aspirin, fibrates, furosemide, statins, insulin), interventions (continuous renal replacement therapy, mechanical ventilation), laboratory parameters (Hb, platelets, calcium, potassium, sodium, creatinine, INR, white blood cell), and clinical scores (SAPSII and GCS).

To further evaluate the additional predictive value of HGI for the risk of PSD, we calculated the area under the receiver operating characteristic (AUC) curve to quantify the predictive power of each model. The DeLong method was used to assess the change in AUC after adding HGI. Then, the net reclassification index (NRI) and the integrated discriminant index (IDI) were calculated to assess the risk reclassification ability of HGI.

To examine potential nonlinearity, HGI was analyzed as a continuous variable using restricted cubic splines (RCS). If nonlinearity was identified, a recursive algorithm was used to determine the inflection point between HGI and PSD. To further evaluate the potential nonlinear association, a two‐piecewise logistic regression model was applied on each side of the inflection point.

Additionally, subgroup analyses were performed by age group (< 70 vs. ≥ 70 years), sex (male vs. female), history of diabetes, hypertension, sepsis, and invasive mechanical ventilation. All analyses were conducted with R (v4.5.0). *p* < 0.05 was considered statistically significant.

## 3 Results

### 3.1 Baseline Characteristics

A total of 10,314 individuals with stroke were identified in the MIMIC‐IV database. Finally, 2247 patients with HGI met the inclusion criteria. The median age among the participants was 69.80 years, with a range from 59.19 to 79.64 years, and 52.6% were male. According to tertiles of HGI, participants were divided into three groups: T1 (HGI ≤ −3.92, *n* = 749), T2 (−3.92 < HGI ≤ 0.14, *n* = 749), and T3 (HGI > 0.14, *n* = 749). Baseline characteristics for participants are presented in Table 1. Compared to patients with low HGI, those with high HGI showed higher prevalence of hypertension and diabetes, higher use of insulin, statins, aspirin, furosemide, and fibrates, and higher HbA1c and platelet levels. Conversely, individuals with low HGI (T1) had increased incidence of delirium, longer ICU‐LOS, and higher heart rate, FPG, and white blood cell counts. Additionally, the GCS scores in individuals with low HGI were lower than those with middle and high HGI, while the GCS scores in T2 and T3 were consistent (both 15.00). Red blood cells and Hb levels were highest in patients with middle HGI (Table 1). As shown in Table S4, patients included in the final analysis had more severe clinical profiles than those excluded due to missing HGI data.

**TABLE 1:** Baseline characteristics of patients stratified by HGI tertiles.

Characteristic	*N* ^a^	Overall	T1	T2	T3	*p*‐value^c^
		*N* = 2247^b^	*N* = 749^b^	*N* = 749^b^	*N* = 749^b^	
Gender, *n* (%)	2247					0.558
Female		1065 (47.40%)	367 (49.00%)	348 (46.46%)	350 (46.73%)	
Male		1182 (52.60%)	382 (51.00%)	401 (53.54%)	399 (53.27%)	
Race, *n* (%)	2247					< 0.001
Black		245 (10.90%)	57 (7.61%)	70 (9.35%)	118 (15.75%)	
Others		598 (26.61%)	218 (29.11%)	181 (24.17%)	199 (26.57%)	
White		1404 (62.48%)	474 (63.28%)	498 (66.49%)	432 (57.68%)	
Admission age (years)	2247	69.80 (59.19, 79.64)	67.96 (55.88, 78.95)	70.44 (60.22, 80.09)	70.25 (60.90, 79.81)	< 0.001
Hypertension, *n* (%)	2247					< 0.001
No		1224 (54.47%)	485 (64.75%)	434 (57.94%)	305 (40.72%)	
Yes		1023 (45.53%)	264 (35.25%)	315 (42.06%)	444 (59.28%)	
Sepsis, *n* (%)	2247					0.116
No		1663 (74.01%)	540 (72.10%)	574 (76.64%)	549 (73.30%)	
Yes		584 (25.99%)	209 (27.90%)	175 (23.36%)	200 (26.70%)	
Diabetes, *n* (%)	2247					< 0.001
No		1494 (66.49%)	613 (81.84%)	602 (80.37%)	279 (37.25%)	
Yes		753 (33.51%)	136 (18.16%)	147 (19.63%)	470 (62.75%)	
Invasive_mv, *n* (%)	2247					0.55
No		1930 (85.89%)	635 (84.78%)	649 (86.65%)	646 (86.25%)	
Yes		317 (14.11%)	114 (15.22%)	100 (13.35%)	103 (13.75%)	
Noninvasive mv, *n* (%)	2247					0.065
No		2226 (99.07%)	744 (99.33%)	745 (99.47%)	737 (98.40%)	
Yes		21 (0.93%)	5 (0.67%)	4 (0.53%)	12 (1.60%)	
CRRT flag, *n* (%)	2247					> 0.999
No		2233 (99.38%)	745 (99.47%)	744 (99.33%)	744 (99.33%)	
Yes		14 (0.62%)	4 (0.53%)	5 (0.67%)	5 (0.67%)	
Aspirin, *n* (%)	2247					< 0.001
No		692 (30.80%)	279 (37.25%)	228 (30.44%)	185 (24.70%)	
Yes		1555 (69.20%)	470 (62.75%)	521 (69.56%)	564 (75.30%)	
Fibrate, *n* (%)	2247					0.034
No		2207 (98.22%)	740 (98.80%)	739 (98.66%)	728 (97.20%)	
Yes		40 (1.78%)	9 (1.20%)	10 (1.34%)	21 (2.80%)	
Furosemide, *n* (%)	2247					< 0.001
No		1459 (64.93%)	512 (68.36%)	510 (68.09%)	437 (58.34%)	
Yes		788 (35.07%)	237 (31.64%)	239 (31.91%)	312 (41.66%)	
Statin, *n* (%)	2247					< 0.001
No		611 (27.19%)	275 (36.72%)	211 (28.17%)	125 (16.69%)	
Yes		1636 (72.81%)	474 (63.28%)	538 (71.83%)	624 (83.31%)	
Insulin, *n* (%)	2247					< 0.001
No		540 (24.03%)	201 (26.84%)	234 (31.24%)	105 (14.02%)	
Yes		1707 (75.97%)	548 (73.16%)	515 (68.76%)	644 (85.98%)	
Delirium, *n* (%)	2247					< 0.001
No		1540 (68.54%)	459 (61.28%)	554 (73.97%)	527 (70.36%)	
Yes		707 (31.46%)	290 (38.72%)	195 (26.03%)	222 (29.64%)	
LOS ICU (day)	2247	2.95 (1.81, 5.08)	3.38 (1.93, 5.89)	2.78 (1.73, 4.80)	2.81 (1.75, 4.71)	< 0.001
GCS score	2247	15.00 (14.00, 15.00)	14.00 (13.00, 15.00)	15.00 (14.00, 15.00)	15.00 (14.00, 15.00)	0.006
SAPSII score	2247	29.00 (22.00, 36.00)	28.00 (22.00, 36.00)	28.00 (22.00, 36.00)	29.00 (23.00, 37.00)	0.209
SPO2 (%)	2247	97.00 (95.00, 99.00)	97.00 (96.00, 99.00)	97.00 (95.00, 99.00)	97.00 (95.00, 99.00)	0.314
RR (breaths/min)	2247	18.00 (15.00, 21.00)	18.00 (15.00, 21.00)	18.00 (15.00, 21.00)	18.00 (15.00, 21.00)	0.736
HR (beats/min)	2247	80.00 (70.00, 92.00)	82.00 (71.00, 94.00)	79.00 (69.00, 91.00)	80.00 (69.00, 92.00)	0.009
FBG (mg/dL)	2247	117.00 (99.00, 148.00)	129.00 (110.00, 157.00)	108.00 (96.00, 126.00)	115.00 (95.00, 169.00)	< 0.001
Hemoglobin (g/dL)	2247	12.20 (10.60, 13.70)	12.20 (10.70, 13.70)	12.40 (10.80, 13.80)	12.00 (10.50, 13.60)	0.043
HbA1c (%)	2247	5.70 (5.40, 6.40)	5.30 (5.00, 5.60)	5.70 (5.50, 5.90)	6.70 (6.00, 8.00)	< 0.001
Platelet (10^9/L)	2247	211.00 (166.00, 260.00)	211.00 (168.00, 259.00)	209.00 (164.00, 261.00)	214.00 (165.00, 262.00)	0.74
RBC (10^6/µL)	2247	4.08 (3.57, 4.54)	4.03 (3.51, 4.48)	4.12 (3.65, 4.54)	4.08 (3.59, 4.60)	0.032
WBC (10^3/µL)	2247	9.30 (7.30, 12.10)	9.80 (7.70, 12.70)	8.90 (7.00, 11.40)	9.20 (7.20, 11.90)	< 0.001
Calcium (mg/dL)	2247	8.80 (8.40, 9.20)	8.80 (8.40, 9.10)	8.90 (8.50, 9.20)	8.90 (8.40, 9.20)	0.165
Potassium (mEq/L)	2247	4.00 (3.70, 4.30)	4.00 (3.70, 4.30)	4.00 (3.70, 4.30)	4.00 (3.70, 4.40)	0.454
Sodium (mEq/L)	2247	139.00 (137.00, 141.00)	139.00 (136.00, 141.00)	139.00 (137.00, 141.00)	139.00 (137.00, 141.00)	0.461
Creatinine (mg/dL)	2247	0.90 (0.70, 1.10)	0.90 (0.70, 1.10)	0.90 (0.70, 1.10)	0.90 (0.70, 1.20)	< 0.001

Abbreviations: CRRT, continuous renal replacement therapy; FBG, fasting blood glucose; GCS, Glasgow Coma Scale; HbA1c, hemoglobin A1c; HGI, hemoglobin glycation index; HR, heart rate; ICU, intensive care unit; INR, international normalized ratio; MV, mechanical ventilation; RBC, red blood cell; RR, respiratory rate; SAPSII, Simplified Acute Physiology Score II; SPO2, pulse blood oxygen saturation; WBC, white blood cell.

A: Number of non‐missing observations used for the variable.

B: Median (25th percentile, 75th percentile) for continuous variables; n (%) for categorical variables.

C: P‐values derived from chi‐square test for categorical variables and Kruskal‐Wallis test for continuous variables.

### 3.2 Association Between HGI and PSD

Baseline comparisons revealed that the mortality rate was lowest in the T2 group (−3.92 < HGI ≤ 0.14). Therefore, we developed logistic regression models using the T2 group as the reference group to ascertain the association between HGI and the primary outcome. When HGI was modeled as a continuous variable, in the unadjusted analysis (Model 1), a one‐unit increase in HGI corresponded to a 14.5% decrease in the risk of PSD among stroke patients (OR = 0.855, 95% CI: 0.782–0.931, *p* < 0.001). Following preliminary adjustment for sex and age in Model 2, the negative association remained significant, and every one‐unit increase in HGI was associated with a 13.3% reduction in the risk of PSD (OR = 0.867, 95% CI: 0.779–0.961, *p* = 0.007). However, after further adjustment for potential confounders in Model 3, this negative association was no longer statistically significant (OR = 0.887, 95% CI: 0.757–1.034, *p* = 0.130).

When HGI was analyzed as a categorical variable in Model 1, the T1 group was significantly associated with a 79.5% higher risk of PSD during ICU stay compared to T2 (OR = 1.795, 95% CI: 1.442–2.238, *p* < 0.001). In Model 2, the T1 group was significantly associated with an 84.3% higher risk of PSD compared to T2 (OR = 1.843, 95% CI: 1.425–2.390, *p* < 0.001). In Model 3, the T1 group was still significantly associated with a 69.7% higher risk of PSD compared to T2 (OR = 1.697, 95% CI: 1.210–2.386, *p* = 0.002) (Table 2).

**TABLE 2:** LR analysis of the association between HGI and PSD risk in stroke patients.

Characteristic	Model 1		Model 2		Model 3	
	OR (95% CI)	*p*‐value	OR (95% CI)	*p*‐value	OR (95% CI)	*p*‐value
HGI (continuous)	0.855 (0.782, 0.931)	< 0.001	0.867 (0.779, 0.961)	0.007	0.887 (0.757, 1.034)	0.130
group						
T2	—		—		—	
T3	1.197 (0.954, 1.502)	0.12	1.225 (0.941, 1.597)	0.133	1.233 (0.853, 1.786)	0.266
T1	1.795 (1.442, 2.238)	< 0.001	1.843 (1.425, 2.390)	< 0.001	1.697 (1.210, 2.386)	0.002

*Note*: Model 1 represents the unadjusted model. Model 2 adjusts for gender and age. Model 3 further adjusts based on Model 2 for race, vital signs (SPO2, RR, HR), comorbidities (hypertension, sepsis, diabetes), medication use (aspirin, fibrate, furosemide, statin, insulin), special treatments (CRRT, MV), laboratory parameters (hemoglobin, platelet, calcium, potassium, sodium, creatinine, WBC, INR), and clinical scores (SAPS II, GCS).

Abbreviations: CRRT, continuous renal replacement therapy; GCS, Glasgow Coma Scale; HGI, hemoglobin glycation index; HR, heart rate; INR, international normalized ratio; MV, mechanical ventilation; RR, respiratory rate; SAPS II, Simplified Acute Physiology Score II; SPO2, pulse blood oxygen saturation; WBC, white blood cell.

### 3.3 Nonlinear Relationship

**FIGURE 2:**
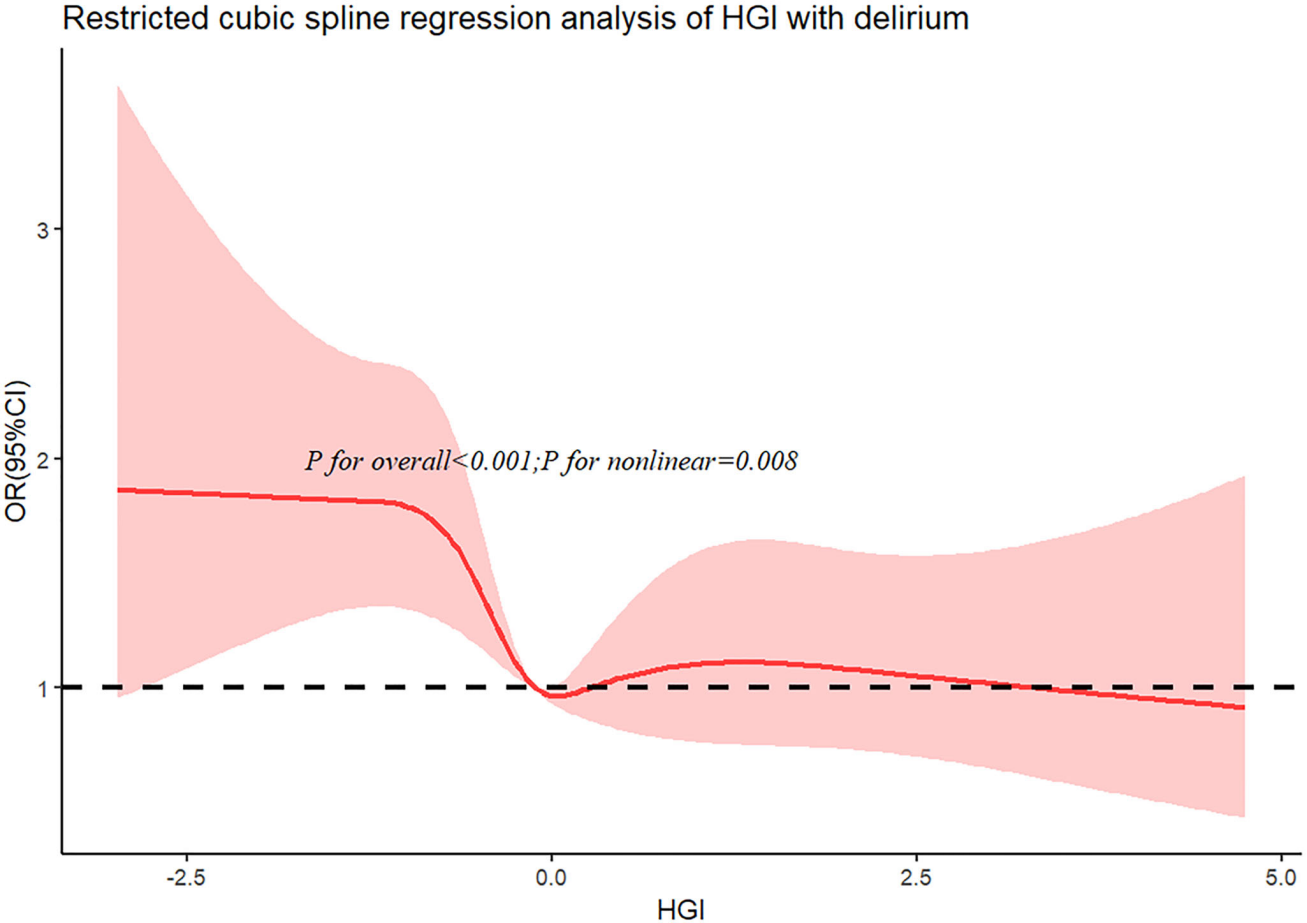
RCS of HGI with delirium occurrence during ICU stay in stroke patients. Abbreviations: HGI, hemoglobin glycation index; ICU, intensive care unit; RCS, restricted cubic spline.

RCS analysis indicated a significant nonlinear association between HGI and PSD risk in stroke patients (*p* for overall association < 0.001, *p* for nonlinearity = 0.008), indicating that the effect of HGI on PSD risk was not just a linear trend but followed a specific trend across HGI levels. To further explore this nonlinear relationship, logistic regression and two‐piecewise logistic regression models (both with log‐likelihood ratio *p* < 0.05) were applied, identifying an inflection point for the risk of PSD at HGI = −0.11. When HGI < −0.11, a one‐unit increase in HGI corresponded to a 30.9% lower risk of PSD (OR = 0.691, 95% CI: 0.554–0.859, *p* < 0.001). When HGI > −0.11, the results were not statistically significant (OR = 0.976, 95% CI: 0.843–1.125, *p* = 0.745) (Figure 2 and Table 3).

**TABLE 3:** Threshold effect analysis of HGI on the PSD risk in stroke patients.

Standard linear regression model	0.864 (0.777, 0.957) 0.006
Two‐stage regression models	
Inflection point (K)	−0.11
< K	0.691 (0.554, 0.859) < 0.001
> K	0.976 (0.843, 1.125) 0.745
Log·likelihood ratio test	< 0.001

### 3.4 Incremental Predictive Value of HGI for the Risk of PSD

We compared the predictive ability of the model based on SAPSII alone and the model based on SAPSII combined with HGI for PSD. The predictive ability of each model was quantified by calculating the AUC. The AUC increased from 0.696 for the model based on SAPSII alone to 0.706 for the model based on HGI combined with SAPSII, with a ΔAUC of 0.010, which was statistically significant (*p* < 0.001). The NRI and IDI were further calculated to assess the risk reclassification ability of the models. The NRI of the model based on HGI combined with SAPSII increased to 0.3105 (*p* < 0.001) and the IDI to 0.0139 (*p* < 0.001), both statistically significant. These results suggested that incorporating HGI helped improve the ability of the predictive model to reclassify patients at high risk of PSD (Table 4).

**TABLE 4:** Incremental predictive value of HGI added to SAPSII for PSD.

	Predictive model	*C*‐statistic	*p*‐value	NRI	*p*‐value	IDI	*p*‐value
Delirium	SAPSII	0.696	—	—	—	—	—
	SAPSII + HGI	0.706	< 0.001	0.3105	< 0.001	0.0139	< 0.001

### 3.5 Subgroup Analyses

**FIGURE 3:**
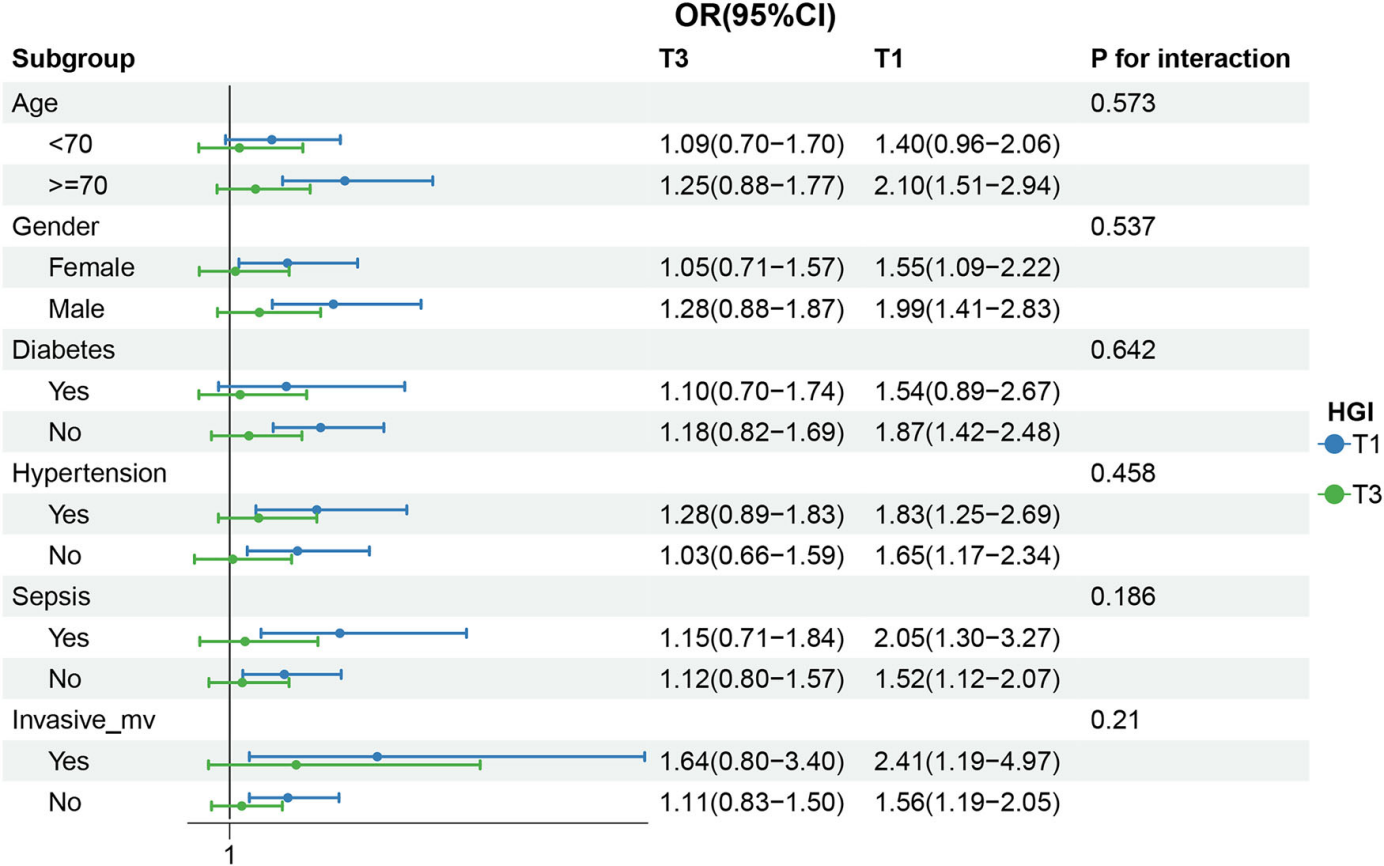
Forest plots of stratified analyses of HGI and delirium occurrence during ICU stay in stroke patients. T1, low HGI group; T2, middle HGI group; T3, high HGI group. Abbreviation: ICU, intensive care unit.

To further assess whether the association between HGI levels and the risk of PSD differed across clinical subgroups, we performed subgroup analyses according to age, sex, hypertension, diabetes, sepsis, and invasive mechanical ventilation. The results showed that in old stroke patients, the association between low HGI and the risk of PSD was more evident. The association between low HGI and the risk of PSD was observed in both males and females. Among patients with or without hypertension, sepsis, or diabetes, patients with low HGI had a higher risk of PSD compared to the reference group. Similarly, in patients who received or did not receive invasive mechanical ventilation, patients with low HGI also exhibited a higher risk of PSD, with a greater magnitude of risk increase in patients receiving invasive mechanical ventilation. Interaction analyses were performed to assess heterogeneity across subgroups (all *p* for interaction > 0.05). The results indicated that the association did not depend on age, sex, presence of diabetes, hypertension, sepsis, or invasive mechanical ventilation. These findings suggested that the association of HGI with the risk of PSD was consistent across different clinical subgroups (Figure 3).

## 4 Discussion

As far as we know, this study is the first to comprehensively assess the association between HGI and the risk of PSD. Our study revealed a significant nonlinear association between HGI and the risk of PSD. These findings suggest that HGI levels below a certain inflection point may be significantly associated with the risk of PSD, providing guidance for clinical intervention strategies for reducing the risk of PSD.

### 4.1 Association Between HGI and PSD and Potential Mechanisms

HbA1c is produced via the nonenzymatic reaction between Hb A1 and glucose in the intracellular environment, and the actual HbA1c level may differ from the predicted value (Mukherjee et al. 2024). However, glycated Hb levels are influenced by various physiological and pathological factors. HGI is estimated to reflect the difference between observed and predicted HbA1c, serving as a measure of glycemic control status. A low HGI value indicates that the observed HbA1c is lower compared to predicted values, which may reflect shorter red blood cell lifespan, reduced glycation, or faster erythrocyte turnover and clearance. Previous studies have linked HGI to differences in red blood cell survival and individual glycation tendencies (Hsia et al. 2020; Cohen and Smith 2008). Given this heterogeneity of erythrocyte metabolism and glycation, patients’ true glycemic exposure and acute glucose fluctuations may be masked by low glycation levels, leading to unrecognized metabolic disturbances that further disrupt cerebral metabolic homeostasis. Specifically, it is possible that glucose metabolic abnormalities related to low HGI could suppress cholinergic neuronal activity, with a consequent decrease in acetylcholine levels. Studies have reported an association between reduced cholinergic activity and delirium, although direct evidence linking HGI to this pathway is lacking (Maldonado 2017; Shafi et al. 2017). Furthermore, low HGI may be accompanied by insulin metabolic dysregulation, which has been linked to amyloid‐β metabolism in the brain (Geng et al. 2024). It is thus hypothesized that extracellular Aβ deposition could trigger intracellular tau phosphorylation, with potential adverse effects on synaptic function and cognition (Bloom 2014; Ishibashi et al. 2024). Additionally, the brain, as an energy‐intensive organ, depends on a continuous glucose supply. When HGI is too low, short‐term glucose fluctuations combined with poor long‐term glucose control may theoretically result in insufficient energy supply to neural cells. Cerebral ischemia and hypoxia might contribute to mitochondrial dysfunction and lactate accumulation, potentially impairing brain regions closely related to cognitive function. This energy metabolic disturbance directly disrupts neuronal function, which is amplified in the context of poststroke brain injury (Li et al. 2025; Meerwaldt et al. 2023). Finally, poststroke patients experience stress‐induced inflammatory responses, and glucose metabolic disturbances further stimulate the immune system to secrete proinflammatory cytokines encompassing IL‐1β and TNF‐α. These cytokines pass through the blood–brain barrier and activate microglia cells, triggering central inflammation and damaging synaptic function and brain network connectivity, which are closely related to the characteristic fluctuations in consciousness and cognitive dysfunction observed in delirium patients (van Keulen et al. 2018; Inouye et al. 2014; Leonard et al. 2015). It should be emphasized that the above mechanisms are primarily inferred from previous studies. This study did not directly measure the relevant biological pathways.

Delirium is common in stroke patients. One study has reported that the incidence of PSD during ICU stay can be as high as approximately 44% (Lawson et al. 2025). Its occurrence and development are closely linked to glucose fluctuations (Lin et al. 2021). Several cohort studies have supported the association between glucose variability and adverse clinical results. In a large cohort study, among individuals with diabetes receiving coronary artery bypass graft surgery with cardiopulmonary support, overly strict glucose control (< 7.8 mmol/L) and greater glucose variability are independently correlated with unfavorable outcomes during hospital stay (You et al. 2023). Similarly, among general ICU populations, glucose variability has been regarded as an independent predictor of both in‐hospital mortality and poor prognosis (Kulkarni et al. 2019). Therefore, extending these well‐established findings from cardiovascular disease and ICU populations to those with stroke to further estimate the association between HGI and PSD, a neurocognitive outcome, has clear epidemiological significance and practical value.

### 4.2 Study Significance and Limitations

This study indicates that HGI, reflecting individual differences in long‐term glycemic exposure and glycation tendency, is significantly associated with the risk of PSD during ICU stays in critically ill stroke patients. PSD is a frequent but often clinically overlooked acute brain dysfunction. HGI, a simple indicator that can be calculated from routine glucose and HbA1c data without additional testing, provides clinicians with a rapid method to identify patients at high risk. Moreover, a single glucose measurement cannot reflect long‐term metabolic status, whereas HGI can capture individual glycemic vulnerability in stroke patients who experience metabolic stress. Incorporating HGI into early stroke assessment may help optimize monitoring, increase vigilance, and guide personalized metabolic management and neuroprotective strategies, potentially improving long‐term outcomes.

However, there are some limitations in this study. First of all, it is a retrospective study based on ICU electronic medical records at a single center, making it difficult to establish causality. Selection bias may exist since patients with missing data on HGI were excluded. The included patients had more severe illness and higher mortality rates, indicating that our findings on the association between HGI and the risk of PSD are primarily applicable to higher‐risk stroke patients and may not be applicable to those with milder strokes. Although we adjusted for multiple clinical variables and conducted subgroup analyses, residual confounding may still exist. For example, medication use, mechanical ventilation parameters, and baseline cognitive function may influence delirium incidence and are difficult to fully control. Moreover, other clinically important confounders associated with PSD, including stroke subtype, stroke severity indices such as NIHSS, sedative/antipsychotic medication use, alcohol withdrawal history, and ICU sedation depth, were not available in the MIMIC‐IV database and could not be adjusted for in our analyses. Although we incorporated available proxy variables including GCS score, SAPSII, and mechanical ventilation status into our models, these proxies cannot fully substitute for the aforementioned missing information, and residual confounding may persist. Furthermore, the inflection point (HGI = −0.11) identified in this study was derived from this single‐center cohort. Whether this threshold can be generalized to broader populations needs to be further validated in multicenter prospective studies. Second, the sample size is limited and predominantly includes critically ill stroke patients, which may not represent the general stroke population. Therefore, data from expanded cohort studies are desired to corroborate our findings. Third, HGI was calculated based on HbA1c and initial admission glucose, and we could not confirm whether glucose measurements were fasting or affected by stress, which may introduce bias. HGI is a surrogate marker of glucose variability, but this study did not include continuous glucose monitoring data during ICU stay. Hence, the role of short‐term glucose fluctuations in delirium could not be assessed. Additionally, the database lacks certain key variables, such as quantitative imaging of brain injury and changes in inflammatory biomarkers. Hence, we cannot explore the biological mechanisms linking HGI to cerebral metabolic dysfunction. Finally, due to recording limitations of the database, we could not precisely determine the time window of delirium onset or its dynamic relationship with glucose changes, nor clarify whether stroke subtype, lesion location, and volume modulate this relationship, which somewhat limits the clinical interpretability of our findings.

## 5 Conclusions

This study reveals that HGI is a potential auxiliary reference indicator for assessing the risk of PSD during ICU stays in critically ill stroke individuals. In addition, nonlinear associations between HGI levels and PSD risk are observed. HGI may be helpful for identifying stroke patients likely to develop PSD during ICU stay and provide clues for metabolic management. However, these findings need to be further validated in prospective multicenter cohorts.

## Author Contributions

**Xu Yang**: data curation, Writing – original draft. **Yong Yin**: writing – review and editing. **Yuwen Wang**: conceptualization, methodology, software.

## Funding

This work was funded by the Yunnan University Medical Research Foundation (No. YDYXJJ2025‐0018 and No. YDYXJJ2025‐0002).

## Ethics Statement

Due to the retrospective nature of this study and the de‐identified, publicly accessible MIMIC‐IV database, the Institutional Review Board (IRB) at the Massachusetts Institute of Technology (MIT) waived the requirement for informed consent. Additionally, since the data are de‐identified and publicly available, no additional ethical approval is required for the use of MIMIC‐IV data in China.

## Conflicts of Interest

The authors declare no conflicts of interest.

## Supporting information




**Supplementary Table S1** Missingness of variables extracted from the MIMiC‐IV cohort before multiple imputation.
**Supplementary Table S2** Regression coefficients for the prediction of HbA1c from FPG
**Supplementary Table S3** Multicollinearity analysis VIF values.
**Supplementary Table S4** Comparison of Baseline Characteristics Between Included and Excluded Patients.

## Data Availability

The data presented in this study are openly available in the MIMIC‐IV (v3.1) at: https://physionet.org/content/mimiciv/3.1/.
