# Supplementary material for: Association Between the Hemoglobin Glycation Index and the Risk of Delirium in Stroke Patients Admitted to the Intensive Care Unit: A Retrospective Study Based on the MIMIC‐IV Database
Source: Brain Behav. 2026 Sep 23;16(9):e71768. doi: 10.1002/brb3.71768 (PMC13601751; doi:10.1002/brb3.71768)
Supplement: Supplementary file 1 [file BRB3-16-e71768-s001.docx]

**Supplementary Materials**

**Supplementary Table S1** Missingness of variables extracted from the MIMiC-IV cohort before multiple imputation.

| Total analytic sample: N = 2,247. Variables with >20% missingness were excluded from the final multivariable models; retained covariates with missing values were handled using multiple imputation. | | | | | | |
| --- | --- | --- | --- | --- | --- | --- |
| Panel | Variable | Definition/label | Non-missing, n | Missing, n | Missing, % | Analysis decision |
| Retained | subject_id | Subject identifier | 2,247 | 0 | 0.00 | Included; complete |
| Retained | invasive_mv | Invasive mechanical ventilation | 2,247 | 0 | 0.00 | Included; complete |
| Retained | noninvasive_mv | Noninvasive mechanical ventilation | 2,247 | 0 | 0.00 | Included; complete |
| Retained | crrt_flag | Continuous renal replacement therapy | 2,247 | 0 | 0.00 | Included; complete |
| Retained | status_30d | 30-day mortality status | 2,247 | 0 | 0.00 | Included; complete |
| Retained | time_30d | Follow-up time for 30-day mortality | 2,247 | 0 | 0.00 | Included; complete |
| Retained | status_90d | 90-day mortality status | 2,247 | 0 | 0.00 | Included; complete |
| Retained | time_90d | Follow-up time for 90-day mortality | 2,247 | 0 | 0.00 | Included; complete |
| Retained | status_1year | 1-year mortality status | 2,247 | 0 | 0.00 | Included; complete |
| Retained | time_1year | Follow-up time for 1-year mortality | 2,247 | 0 | 0.00 | Included; complete |
| Retained | dementia | Dementia | 2,247 | 0 | 0.00 | Included; complete |
| Retained | hypertension | Hypertension | 2,247 | 0 | 0.00 | Included; complete |
| Retained | schizophrenia | Schizophrenia | 2,247 | 0 | 0.00 | Included; complete |
| Retained | sepsis | Sepsis | 2,247 | 0 | 0.00 | Included; complete |
| Retained | diabetes | Diabetes mellitus | 2,247 | 0 | 0.00 | Included; complete |
| Retained | gender | Sex | 2,247 | 0 | 0.00 | Included; complete |
| Retained | race | Race/ethnicity | 2,247 | 0 | 0.00 | Included; complete |
| Retained | admission_age | Age at hospital admission | 2,247 | 0 | 0.00 | Included; complete |
| Retained | gcs | Glasgow Coma Scale score | 2,247 | 0 | 0.00 | Included; complete |
| Retained | sapsii | SAPS II score | 2,247 | 0 | 0.00 | Included; complete |
| Retained | spo2 | Peripheral oxygen saturation | 2,247 | 0 | 0.00 | Included; complete |
| Retained | hr | Heart rate | 2,247 | 0 | 0.00 | Included; complete |
| Retained | aspirin | Aspirin use | 2,247 | 0 | 0.00 | Included; complete |
| Retained | fibrate | Fibrate use | 2,247 | 0 | 0.00 | Included; complete |
| Retained | furosemide | Furosemide use | 2,247 | 0 | 0.00 | Included; complete |
| Retained | statin | Statin use | 2,247 | 0 | 0.00 | Included; complete |
| Retained | insulin | Insulin use | 2,247 | 0 | 0.00 | Included; complete |
| Retained | delirium | Delirium during ICU stay | 2,247 | 0 | 0.00 | Included; complete |
| Retained | fbg | Fasting blood glucose | 2,247 | 0 | 0.00 | Included; complete |
| Retained | hba1c | Glycated hemoglobin | 2,247 | 0 | 0.00 | Included; complete |
| Retained | los_icu | Length of ICU stay | 2,246 | 1 | 0.04 | Multiple imputation |
| Retained | rr | Respiratory rate | 2,232 | 15 | 0.67 | Multiple imputation |
| Retained | first_hemoglobin | Hemoglobin | 2,135 | 112 | 4.98 | Multiple imputation |
| Retained | first_platelet | Platelet count | 2,134 | 113 | 5.03 | Multiple imputation |
| Retained | first_rbc | Red blood cell count | 2,136 | 111 | 4.94 | Multiple imputation |
| Retained | first_wbc | White blood cell count | 2,135 | 112 | 4.98 | Multiple imputation |
| Retained | first_potassium | Potassium | 2,150 | 97 | 4.32 | Multiple imputation |
| Retained | first_sodium | Sodium | 2,155 | 92 | 4.09 | Multiple imputation |
| Retained | first_creatinine | Creatinine | 2,151 | 96 | 4.27 | Multiple imputation |
| Retained | first_calcium | Calcium | 1,984 | 263 | 11.7 | Multiple imputation |
| Retained | first_inr | International normalized ratio | 1,922 | 325 | 14.46 | Multiple imputation |
| Excluded | first_ddimer | D-dimer | 33 | 2,214 | 98.53 | Excluded; >20% missing |
| Excluded | first_albumin | Albumin | 606 | 1,641 | 73.03 | Excluded; >21% missing |
| Excluded | first_hdl | High-density lipoprotein | 1,216 | 1,031 | 45.89 | Excluded; >22% missing |
| Excluded | first_ldl | Low-density lipoprotein | 1,219 | 1,028 | 45.75 | Excluded; >23% missing |
| Excluded | first_cholesterol | Total cholesterol | 1,221 | 1,026 | 45.66 | Excluded; >24% missing |
| Excluded | first_triglycerides | Triglycerides | 1,227 | 1,020 | 45.39 | Excluded; >25% missing |
| Excluded | first_alt | Alanine aminotransferase | 1,205 | 1,042 | 46.37 | Excluded; >26% missing |
| Excluded | first_ast | Aspartate aminotransferase | 1,218 | 1,029 | 45.79 | Excluded; >27% missing |

**Supplementary Table S2** Regression coefficients for the prediction of HbA1c from FPG

|  | Estimate | Std. Error | t value | Pr(>\|t\|) |
| --- | --- | --- | --- | --- |
| Intercept | 4.253 | 0.056 | 75.53 | < 0.001 |
| FPG | 0.014 | 0.0004 | 37.17 | < 0.001 |
| R² = 0.381 | | | | |
| Regression equation: HbA1c (predicted) = 0.014 × FPG + 4.253 | | | | |

**Supplementary Table S3** Multicollinearity analysis VIF values.

| Variable | VIF |
| --- | --- |
| group | 1.315119 |
| gender | 1.188492 |
| race | 1.144613 |
| age | 1.615910 |
| hypertension | 1.254288 |
| sepsis | 1.136251 |
| diabetes | 1.327880 |
| aspirin | 1.091957 |
| fibrate | 1.033873 |
| los icu | 1.081054 |
| gcs | 1.100667 |
| sapsii | 1.860624 |
| spo2 | 1.107565 |
| rr | 1.125761 |
| hr | 1.195842 |
| hemoglobin | 1.530190 |
| inr | 1.180127 |
| platelet | 1.106679 |
| wbc | 1.044678 |
| calcium | 1.277639 |
| potassium | 1.170088 |
| sodium | 1.064666 |
| creatinine | 1.265001 |

**Supplementary Table S4** Comparison of Baseline Characteristics Between Included

and Excluded Patients.

| Variable | Non-missing | Overall（N=3080） | Excluded（N=833） | Included（N=2247） | P-value |
| --- | --- | --- | --- | --- | --- |
| Gender,n(%) | 3080 |  |  |  | 0.368 |
| Female |  | 1475 (47.89%) | 410 (49.22%) | 1,065 (47.40%) |  |
| Male |  | 1605 (52.11%) | 423 (50.78%) | 1,182 (52.60%) |  |
| Race,n(%) | 3,080 |  |  |  | <0.001 |
| Black |  | 293 (9.51%) | 48 (5.76%) | 245 (10.90%) |  |
| Others |  | 831 (26.98%) | 233 (27.97%) | 598 (26.61%) |  |
| White |  | 1,956 (63.51%) | 552 (66.27%) | 1,404 (62.48%) |  |
| Admission age(years) | 3,080 | 68.23 (56.89, 78.85) | 63.04 (51.69, 75.16) | 69.80 (59.19, 79.64) | <0.001 |
| Hypertension,n(%) | 3,080 |  |  |  |  |
| No |  | 1,773 (57.56%) | 549 (65.91%) | 1,224 (54.47%) |  |
| Yes |  | 1,307 (42.44%) | 284 (34.09%) | 1,023 (45.53%) |  |
| Sepsis,n(%) | 3,080 |  |  |  | 0.788 |
| No |  | 3,006 (97.60%) | 814 (97.72%) | 2,192 (97.55%) |  |
| Yes |  | 74 (2.40%) | 19 (2.28%) | 55 (2.45%) |  |
| Diabetes,n(%) | 3,080 |  |  |  | <0.001 |
| No |  | 2,222 (72.14%) | 728 (87.39%) | 1,494 (66.49%) |  |
| Yes |  | 858 (27.86%) | 105 (12.61%) | 753 (33.51%) |  |
| Invasive_mv,n(%) | 3,080 |  |  |  | 0.414 |
| No |  | 2,655 (86.20%) | 725 (87.03%) | 1,930 (85.89%) |  |
| Yes |  | 425 (13.80%) | 108 (12.97%) | 317 (14.11%) |  |
| Noninvasive mv,n(%) | 3,080 |  |  |  | 0.368 |
| No |  | 3,054 (99.16%) | 828 (99.40%) | 2,226 (99.07%) |  |
| Yes |  | 26 (0.84%) | 5 (0.60%) | 21 (0.93%) |  |
| Crrt flag,n(%) | 3,080 |  |  |  | — |
| No |  | 0 (0.00%) | 0 (0.00%) | 0 (0.00%) |  |
| Yes |  | 3,080 (100.00%) | 833 (100.00%) | 2,247 (100.00%) |  |
| Aspirin,n(%) | 3,080 |  |  |  | <0.001 |
| No |  | 1,230 (39.94%) | 538 (64.59%) | 692 (30.80%) |  |
| Yes |  | 1,850 (60.06%) | 295 (35.41%) | 1,555 (69.20%) |  |
| Fibrate,n(%) | 3,080 |  |  |  | 0.015 |
| No |  | 3,035 (98.54%) | 828 (99.40%) | 2,207 (98.22%) |  |
| Yes |  | 45 (1.46%) | 5 (0.60%) | 40 (1.78%) |  |
| Furosemide,n(%) | 3,080 |  |  |  | <0.001 |
| No |  | 2,082 (67.60%) | 623 (74.79%) | 1,459 (64.93%) |  |
| Yes |  | 998 (32.40%) | 210 (25.21%) | 788 (35.07%) |  |
| Statin,n(%) | 3,080 |  |  |  | <0.001 |
| No |  | 1,142 (37.08%) | 531 (63.75%) | 611 (27.19%) |  |
| Yes |  | 1,938 (62.92%) | 302 (36.25%) | 1,636 (72.81%) |  |
| Insulin,n(%) | 3,080 |  |  |  | <0.001 |
| No |  | 879 (28.54%) | 339 (40.70%) | 540 (24.03%) |  |
| Yes |  | 2,201 (71.46%) | 494 (59.30%) | 1,707 (75.97%) |  |
| delirium,n(%) | 3,080 |  |  |  | <0.001 |
| No |  | 2,116 (68.70%) | 576 (69.15%) | 1,540 (68.54%) |  |
| Yes |  | 964 (31.30%) | 257 (30.85%) | 707 (31.46%) |  |
| Los icu(day) | 3,079 | 3.04 (1.82, 5.61) | 3.48 (1.83, 6.96) | 2.95 (1.81, 5.08) | <0.001 |
| GCS score | 3,080 | 15.00 (14.00, 15.00) | 15.00 (14.00, 15.00) | 15.00 (14.00, 15.00) | 0.203 |
| SAPSII score | 3,080 | 28.00 (22.00, 36.00) | 27.00 (22.00, 35.00) | 29.00 (22.00, 36.00) | 0.009 |
| SPO2(%) | 3,080 | 97.00 (96.00, 99.00) | 98.00 (96.00, 99.00) | 97.00 (95.00, 99.00) | 0.15 |
| RR(breaths/min) | 3,059 | 18.00 (15.00, 21.00) | 17.00 (15.00, 20.00) | 18.00 (15.00, 21.00) | 0.002 |
| HR(beats/min) | 3,080 | 80.00 (69.00, 92.00) | 79.00 (68.00, 91.00) | 80.00 (70.00, 92.00) | 0.109 |
| FBG(mg/dL) | 3,077 | 116.00(98.00,144.00) | 114.00(98.00,137.00) | 117.00(99.00,148.00) | <0.001 |
| Hemoglobin(g/dL) | 2,917 | 12.20 (10.70, 13.60) | 12.10 (10.90, 13.40) | 12.20 (10.60, 13.70) | 0.407 |
| HbA1c(%) | 2,247 | 5.70 (5.40, 6.40) | NA (NA, NA) | 5.70 (5.40, 6.40) | — |
| Platelet(10^9/L) | 2,915 | 210.00 (166.00, 260.00) | 208.00 (168.00, 259.00) | 211.00 (166.00, 260.00) | 0.749 |
| RBC(10^6/μL) | 2,918 | 4.05 (3.57, 4.51) | 4.00 (3.57, 4.40) | 4.08 (3.57, 4.54) | 0.015 |
| WBC(10^3/μL) | 2,917 | 9.40 (7.30, 12.20) | 9.50 (7.20, 12.40) | 9.30 (7.30, 12.10) | 0.745 |
| Calcium(mg/dL) | 2,755 | 8.80 (8.40, 9.10) | 8.70 (8.20, 9.10) | 8.80 (8.40, 9.20) | <0.001 |
| Potassium(mEq/L) | 2,938 | 4.00 (3.70, 4.30) | 3.90 (3.60, 4.30) | 4.00 (3.70, 4.30) | <0.001 |
| Sodium(mEq/L) | 2,944 | 139.00 (137.00, 141.00) | 139.00 (137.00, 141.00) | 139.00 (137.00, 141.00) | 0.938 |
| Creatinine(mg/dL) | 2,944 | 0.80 (0.70, 1.10) | 0.80 (0.70, 1.10) | 0.80 (0.60, 1.00) | <0.001 |

GCS,glasgow coma scale;SAPSII, simplified acute physiology score;SPO2, pulse blood oxygen saturation;RR, respiratory rate;HR, heart rate;FBG,fasting Blood Glucose;HbA1c, hemoglobin A1c;INR, international normalized ratio; RBC,red blood cell;WBC,white blood cell;CRRT,continuous renal replacement therapy;MV,mechanical ventilation;ICU, intensive care unit;HGI, hemoglobin glycation index.
